# Neck lymph node metastasis detection in patients with differentiated thyroid carcinoma (DTC) in long-term follow-up: a ^131^I-SPECT/CT study

**DOI:** 10.1186/s12885-020-06744-1

**Published:** 2020-03-20

**Authors:** Angela Spanu, Susanna Nuvoli, Andrea Marongiu, Ilaria Gelo, Luciana Mele, Bastiana Piras, Giuseppe Madeddu

**Affiliations:** Unit of Nuclear Medicine, Department of Medical, Surgical and Experimental Sciences, University of Sassari, Viale San Pietro 8, 07100 Sassari, Italy

**Keywords:** Differentiated thyroid carcinoma (DTC), Neck lymph node metastases, Long-term follow-up, 131I-whole body scan (WBS), 131I-SPECT/CT

## Abstract

**Background:**

The identification of neck lymph node (LN) metastases represents a very important issue in the management of patients with differentiated thyroid carcinoma (DTC). To this purpose, in the present study, we used 131I-SPECT/CT as a diagnostic imaging procedure.

**Methods:**

A consecutive series of 224 DTC patients with ascertained neck radioiodine-avid foci at ^131^I-SPECT/CT during long-term follow-up was evaluated. All patients had already undergone total thyroidectomy and radioiodine therapy and had been classified as follows: 62 at high risk (H), 64 at low risk (L) and 98 at very low risk (VL). ^131^I-Whole body scan (WBS) followed by SPECT/CT was performed in all cases.

**Results:**

In the 224 patients, 449 neck iodine avid foci were ascertained at SPECT/CT, while 322 were evidenced at WBS in 165/224 patients. WBS classified as residues 263/322 foci and as unclear 59/322 foci; among the former foci SPECT/CT correctly characterized 8 LN metastases and 3 physiologic uptakes and among the latter, it pinpointed 26 LN metastases, 18 residues, and 15 physiologic uptakes. SPECT/CT also classified 127 foci occult at WBS as 59 LN metastases and 68 residues. Globally, SPECT/CT identified 93 LN metastases in 59 patients (26 H, 20 L, 13 VL), while WBS evidenced 34 in 25 cases. All 13 VL patients, T1aN0M0, 5 of whom with LN near sub-mandibular glands, had thyroglobulin undetectable or < 2.5 ng/ml. Globally, SPECT/CT obtained an incremental value than WBS in 45.5% of patients, a more correct patient classification changing therapeutic approach in 30.3% of cases and identified WBS false-positive findings in 8% of cases.

**Conclusions:**

^131^I-SPECT/CT proved to correctly detect and characterize neck LN metastases in DTC patients in long-term follow-up, improving the performance of planar WBS. SPECT/CT routine use is thus suggested; its role is particularly relevant in patients with WBS inconclusive, VL, T1aN0M0 and with undetectable or very low thyroglobulin levels.

## Background

The neck represents the most frequent site of lymph node (LN) metastases from differentiated thyroid carcinoma (DTC), both macro and not rarely micro metastases; their incidence varies with the type of tumor being a common finding in papillary carcinoma (PC) and also in microcarcinoma, in particular when they are multifocal and with extra-thyroid spread [1]. However, in follicular carcinoma (FC) neck lymph node metastases are much less frequent.

The prognostic importance of regional LN metastases at initial diagnosis of carcinoma is controversial even if it is generally accepted that their presence, especially if in bilateral regions, correlates with tumor recurrences and with adverse prognosis, in particular in older age group [1–3], predicting a higher risk of treatment failure [3]. Moreover, some authors reported a deleterious impact of LN metastases also on patient survival [4, 5], and with worse disease-free rates in patients with laterocervical LN metastases in respect of patients with only central compartment nodes and those cases with no nodal disease [6]. However, these data have not been confirmed in previous studies in which no impact on recurrence or survival was ascertained [7].

Conventional ^131^I-planar whole-body scan (WBS), in association with serum thyroglobulin measurement and radiologic procedures has been considered for many years the routine diagnostic procedure in the protocol of patients thyroidectomized for DTC. However, WBS presents some limitations, such as low sensitivity due to gamma camera low resolution for sub-centimeter lesions, sensitivity values reported in literature ranging between 45 and 75% and specificity values ranging between 90 and 100% [8–13]. The lack of anatomic land markers can also cause difficulties for foci localization. In particular, the presence of numerous sites of radioiodine physiologic uptake in the neck can affect image interpretation making difficult the characterization of radioiodine foci; using WBS it is not easy to distinguish physiologic activity and remnant thyroid tissue from residual/recurrent cancer or lymph node metastases, with unclear or even false-positive results [14–19].

More recently, SPECT/CT presents a higher sensitivity and a better contrast resolution than planar acquisition and, obtaining both functional and anatomic fusion cross-sectional images, it has proved capable of dramatically improving the performance of ^131^I-planar WBS in detecting radioiodine avid foci, increasing sensitivity and accuracy and allowing precise anatomic localization and characterization of the lesions [16, 17, 19–24]. In particular, an accurate characterization of cervical lymph node metastases from normal or benign structures can be difficult without the use of hybrid procedures. The latter can also allow nodal spread detection in patients classified as N0 by histological examination at the surgery and resulted negative also at planar WBS. More recently, SPECT/CT is used in addition to WBS in different clinical situations and, in particular, for staging purpose in post-thyroidectomy phase [25–27] and after the first radioiodine ablation [21, 28–34] as well as in the long-term follow up [16, 19, 35].

Since ^131^I-SPECT/CT has proved superior to planar WBS in detecting radioiodine avid malignant lesions from DTC, thus improving both classification and management of the patients, we further investigated the usefulness of SPECT/CT with diagnostic radioiodine dose in a retrospective study of DTC patients in long-term follow-up. The main object of our research was to evaluate SPECT/CT performance comparatively to WBS in detecting neck lymph node metastases. Moreover, we also considered whether SPECT/CT might have an incremental value than planar WBS contributing to DTC’s current diagnosis and therapeutic protocol reassessment.

## Methods

### Patient population

**Fig. 1:**
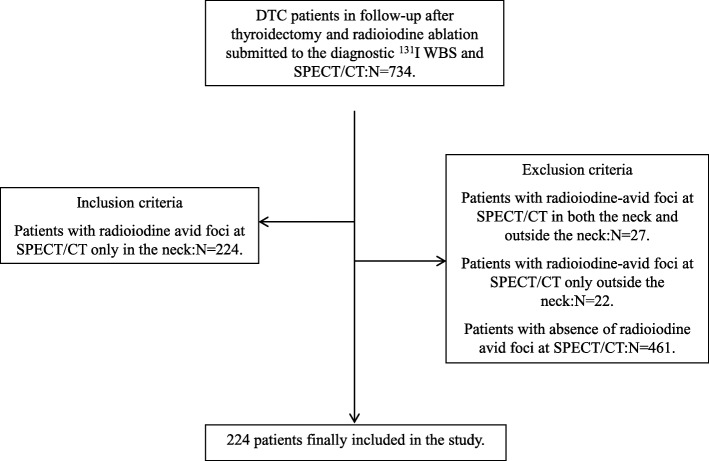
Flow diagram of the patient selection in the study

Among a series of 734 patients with DTC in long-term follow-up after total thyroidectomy and radioiodine therapy, we retrospectively evaluated 224 consecutive cases with ascertained radioiodine avid foci only in the neck at ^131^I-SPECT/CT, which is routinely performed, in our Center since 2006 in all DTC patients during follow up. Inclusion and exclusion criteria are reported in Fig. 1.

As illustrated in Table 1, thyroid PC was identified at the surgery in 211/224 patients, FC in 7 cases and Hürthle cell carcinoma (HCC) in 6 cases. Tumor size was ≤10 mm in 64 cases and > 10 mm in 160 cases; 65 patients were males and 159 females and 77 had an age ≤ 45 years and 147 > 45 years. All patients underwent LN status evaluation with positive loco-regional metastases in 22 cases, 8 patients being T1aN1aM0 and 14 T1aN1bM0, the latter also with palpable laterocervical LN all US positive for malignancy; 59 patients had multifocal/bilateral papillary carcinomas and 39 patients had tumor extra-thyroid extension. Moreover, in accord with the classification of the European Thyroid Cancer Taskforce [36], the patients were classified as being very low risk (VL-unifocal T1 [≤ 1 cm] N0M0 and no extension beyond the thyroid), at low risk (L-T1 [> 1 cm] N0M0, T2N0M0, or multifocal T1N0M0), or high risk (H-any T3 or T4N1M1). Thus, 62 patients were classified at high risk (H), 64 at low risk (L) and 98 at very low risk (VL).

**Table 1:** Demographic and histologic characteristics of the whole group of 224 DTC patients

Gender	159 females; 42 males
Age	≤45 years: 77/224; > 45 years: 147/224
Tumor histology	Papillary carcinoma: 211/224 cases
	Follicular carcinoma: 7/224 cases
	Hürthle cell carcinoma: 6/224 cases
Tumor size	≤10 mm: 64/224
	> 10 mm: 160/224
Tumor structural characteristics	Unifocal carcinoma: 165/224
	Multifocal monolateral papillary carcinoma: 31/224
	Multifocal bilateral papillary carcinoma: 28/224
Risk stratification	High risk: 62/224
	Low risk: 64/224
	Very low risk: 98/224

All patients had a preparation with low-iodine diet for 2 weeks, avoiding iodine-containing medications and underwent diagnostic ^131^I-WBS 48–72 h (h) after an orally administered radioiodine dose of 185 MBq.

One hundred and seventy patients were in hypothyroidism after 4–6 weeks of withdrawal from L-thyroxine or, in some cases who poorly tolerated hypothyroid condition, substituting triiodothyronine to L-thyroxine for 3 weeks; however, 54 patients had received an increment of exogenous thyroid-stimulating hormone (TSH) after recombinant human TSH (rhTSH) stimulation. One hundred and eighty-nine of 224 patients performed one exam each, while 35/224 patients performed two exams each during their follow-up for a total of 259 exams.

### Radioiodine Scintigraphy

Radioiodine planar WBS and SPECT/CT images were acquired by means of a dual-head variable-angle gamma camera complemented with a low dose CT (Infinia Hawkeye 4, GE Healthcare) and equipped with high-energy parallel-hole collimators, setting the ^131^I photon peak (364 Kev) and 20% energy windows. At the beginning, a planar WBS in anterior and posterior views (matrix: 1024 × 256; speed: 5 cm/min for a total time of 30 min) was obtained in all cases with the patient lying in supine position, using a special vacuum cushion to stabilize neck position. SPECT/CT imaging of the neck/chest and other suspected regions was immediately acquired after WBS. SPECT was obtained first, followed by CT scan. SPECT images were acquired covering the region of interest around 360 degrees and using steps of 3 degrees (40 s/step), a 128 × 128 matrix and 1–1.2 zoom factor. CT scan was performed in helical mode at 140 Kev and up to 2.5 mA (total acquisition time: 4–5 min) with the x-ray tube and the detector array rotating together at 2.0 rpm for a 90° L-mode scan. A dedicated software package (Xeleris Workstation; GE Healthcare) was used to process SPECT and CT images. SPECT images were elaborated with the iterative method (OSEM). CT data were reconstructed to obtain cross-sectional attenuation images with a 128 × 128 matrix. Multiplanar (transversal, coronal and sagittal) SPECT and CT images were then merged with each other.

### Data analysis

^131^I-WBS and SPECT/CT images were analyzed independently by three nuclear medicine physicians (AS, SN, and GM) who were aware of the reasons for the examination but were unaware of the results of the other investigations. The three physicians are very experienced; they became interested in 131I-SPECT images in DTC patients in 2006 and 131I-WBS images many years before. Inter-observer variability in this study was very low and disagreements were resolved by consensus

SPECT/CT findings were classified as normal in presence of tracer distribution in normal tissue and physiologic structure or positive in presence of neoplastic and/or unclear foci. ^131^I uptakes were considered neoplastic in the presence of focal areas of increased uptake that are not compatible with physiological storage locations. Neck foci of uptake seen at WBS were considered thyroid remnants when sited in the thyroid bed region and physiologic when sited in the regions of salivary glands, nasopharynx, superior esophagus and trachea. Small-circumscribed areas of increased uptake sited outside the thyroid bed were considered suggestive of lesions. Moreover, planar WBS findings were classified as unclear when it was not easy to give a precise anatomic site or characterization.

SPECT/CT was given an additional value compared to the planar WBS where it provided better identification, correct anatomic localization and interpretation and precise differentiation between neoplastic lesions and normal tissue or physiologic uptake (e.g. cervical metastases vs benign residual tissue). For each patient, focal ^131^I uptake on planar and SPECT/CT images were analyzed for the thyroid bed, lymph nodes in the neck and distant sites in the chest or other regions. Regarding radioiodine uptakes in the neck, when a focus occurred in the follow-up, a change of risk stratification could be considered and a patient from very low risk to low risk could be classified. The results of SPECT/CT were compared with those of planar WBS. SPECT/CT data were confirmed by pathologic findings or by clinical examination, serum thyroglobulin changes and/or radiologic follow-up for at least 12–96 months for recurrences or/and metastases when histology was not available. In patients with positive planar and SPECT/CT findings who underwent radioiodine therapy, post-therapeutic scan findings were also considered.

Before scintigraphy, all patients underwent laboratory tests, such as the measurement of urinary iodine excretion (ioduria) and the assay of serum TSH, thyroglobulin and anti-thyroglobulin antibodies (Ab-Tg) while in hypothyroidism condition or after rh-TSH stimulation.

At scintigraphy, TSH levels were always more than the arbitrary level of 50 μU/ml and those of ioduria were less than 300 μg/L; the cut-off of thyroglobulin was 0.2 ng/ml.

All patients routinely underwent neck ultrasound that we consider one of the more valuable procedures in the follow-up of DTC patients to contribute in identifying neck lymph node metastases, but the data have not enclosed in the text since these are worthwhile-beyond the scope of this study.

All clinical and instrumental examinations were performed in the University Hospital setting as part of the Clinical Care of the thyroid tumor patients and the Unities of Nuclear Medicine and Radiology.

For this type of study, formal consent is not required. The study was performed following the regulations of the Institutional Review Board and in accordance with Helsinki Doctrine. Routinely, before scintigraphy, written informed consent has been obtained by all patients whose data were treated following the local privacy rules and regulations.

### Statistical analysis

Pearson’s chi-squared test was used to compare categorical variables (patient age and tumor size); McNemar test was also used to assess the statistical significance of the differences of per-patient and per-lesion sensitivity between SPECT/CT and planar WBS imaging. The results were considered significant when *P* < 0.05.

## Results

SPECT/CT identified 449 iodine avid neck foci in 224 patients, while planar WBS detected 322 foci in 165 patients all positive at SPECT/CT. The classification of the 449 radioiodine-avid foci evident at SPECT/CT and the corresponding planar WBS findings is illustrated in Table 2.

**Table 2:** Classification of the 449 radioiodine-avid foci evident at SPECT/TC and the corresponding planar WBS findings

SPECT/CT radioiodine foci (*n* = 449)	Corresponding findings at planar WBS
Residues: 338/449	Residues: 252/338
	Unclear: 18/338
	Occult: 68/338
Lymph node metastases: 93/449	Residues: 8/93
	Unclear: 26/93
	Occult: 59/93
Physiologic uptakes: 18/449	Residues: 3/18
	Unclear: 15/18

**Fig. 2:**
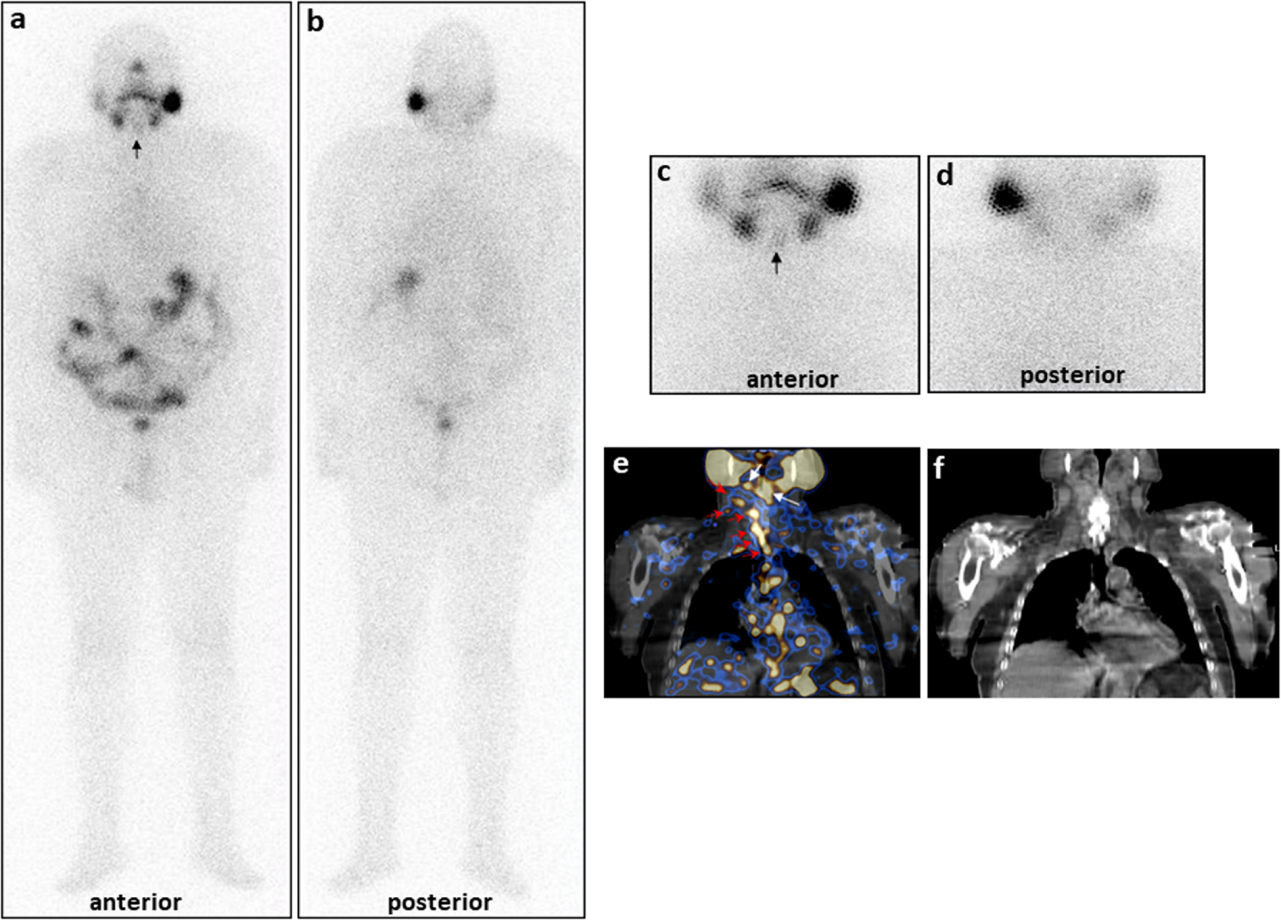
Patient in long-term follow-up with unifocal locally invasive (T4aN0M0) papillary carcinoma already submitted to total thyroidectomy and radioiodine ablation. Diagnostic 131-WBS in both anterior and posterior views (**a**, **b**) and planar anterior and posterior spot views (**c**, **d**) detected a slight radioiodine-avid focus (black arrow) in neck superior middle region classified as residue. SPECT/CT in coronal view (**e**) confirmed this area including two foci (white arrows), and also evidenced numerous foci of elevated radioiodine uptake in the neck right lateral region (red arrows) beginning from the sub-mandibular area as far as jugulum in a vertical line along laterocervical and paratracheal regions. To these foci, lymph nodes corresponded at CT (**f**). Thyroglobulin levels in hypothyroidism were 211 ng/ml; anti-thyroglobulin antibodies were negative. The patient underwent a second surgery and the lymph nodes were ascertained as malignant at histology. Afterward, the patient received two further therapeutic radioiodine doses

Both procedures concordantly classified 252 foci as residues; however, planar WBS wrongly classified or considered unclear 70 foci correctly characterized by SPECT/CT as 18 residues, 34 lymph node metastases and 18 physiologic uptakes (7 of the superior esophagus, 2 of the salivary gland, 6 of the pharynx, 3 of the trachea). Moreover, 127/449 foci were occult at planar WBS (68 residues and 59 lymph node metastases); a case with numerous occult node metastases is illustrated in Fig. 2.

Globally, SPECT/CT identified 93 metastatic foci, 33 of which single, as neck LN metastases in 59 patients, 55 of these with PC, 3 with FC and the remaining one with HCC; 23 patients (39%) had tumor sizes ≤10 mm and 36 cases (61%) > 10 mm (*P* < 0.017). Nineteen cases (32.2%) had an age ≤ 45 years and 40 (67.8%) > 45 years (*P* < 0.0001); 26 patients were H, 20 were L and 13 VL. However, WBS only evidenced 34 foci in 25 patients (12 H, 10 L, 3 VL), in part wrongly classified as residues (8 foci) and in part as unclear (26 foci), the difference in per-patient and per-lesion sensitivity between planar and SPECT/CT being statistically significant (*P* < 0.0001).

A multifocal PC was diagnosed at histology after thyroidectomy in 22/59 patients with neck LN metastases during the follow-up. Of these 22 patients, at surgery, one also had LN metastases and 4 extra-thyroid tumor extension; 4 further cases had both LN metastases and extra-thyroid tumor extension. Moreover, of the remaining 37/59 patients, all of them with unifocal carcinoma, 7/37 PC cases only had palpable neck LN metastases already at surgery; 8/37 (6 PC and 1FC) further cases only had extra-thyroid tumor extension and 1/36 PC had both cervical metastases and extra-thyroid tumor extension. Furthermore, in the other 21/36 patients with localized unifocal thyroid carcinomas (19 PC, 1 FC, 1 HCC) no LN metastasis and/or extra-thyroid tumor extension had been ascertained at surgery.

**Fig. 3:**
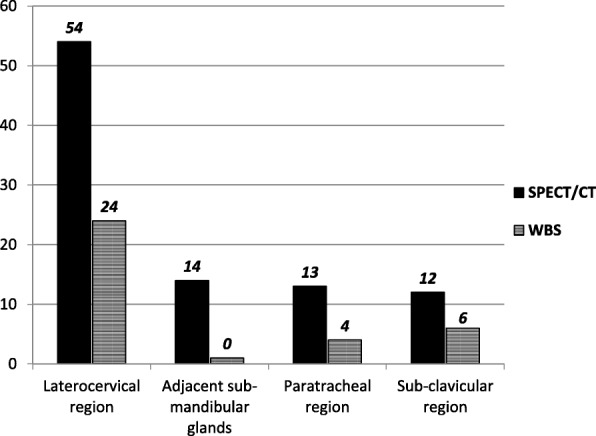
Location of the 93 neck LN metastases ascertained at SPECT/CT and of the 34/93 metastases also evidenced at WBS

**Fig. 4:**
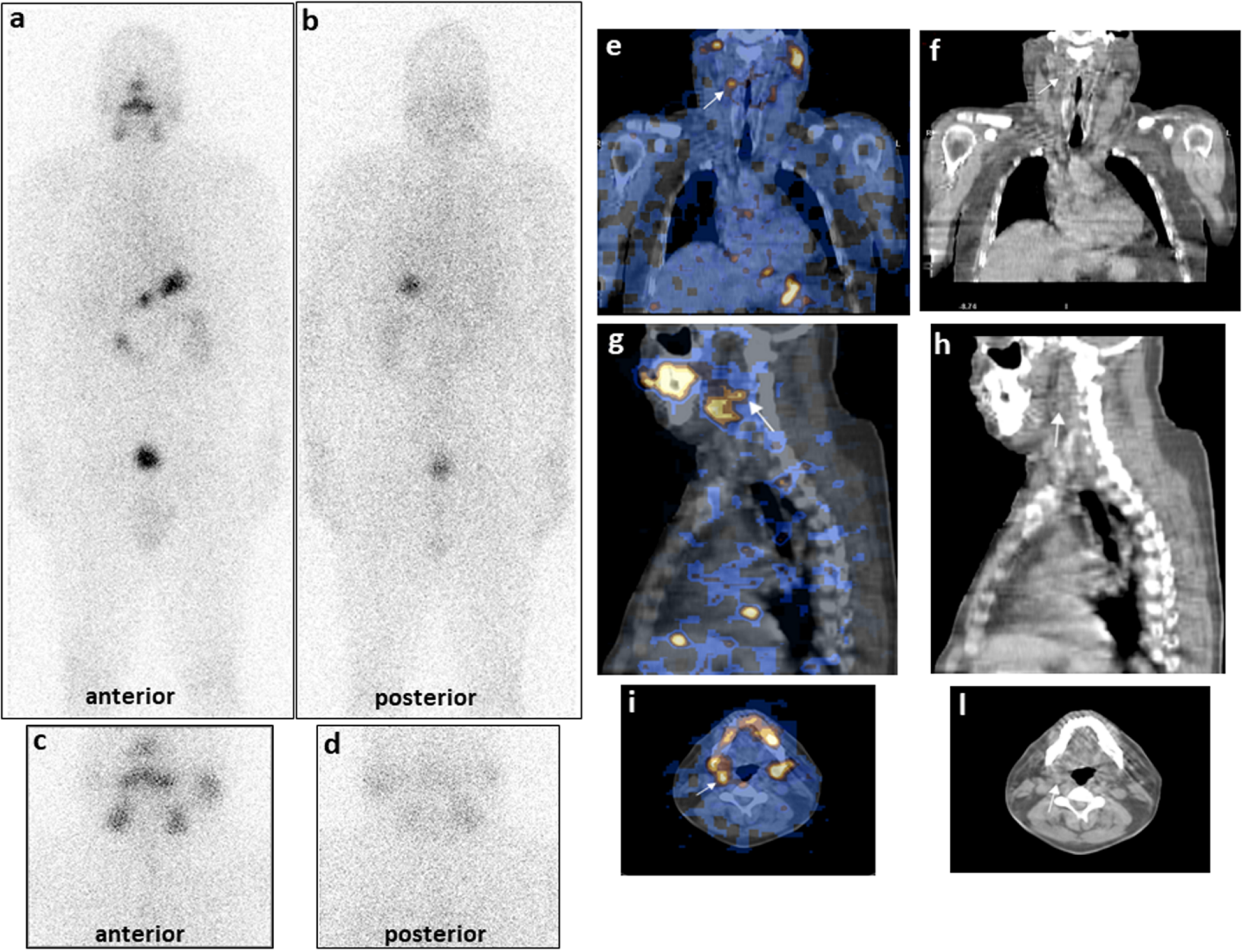
A patient in long-term follow-up affected by unifocal papillary carcinoma with extra-thyroid tumor extension (T4aN0M0) already submitted to total thyroidectomy and radioiodine ablation. Diagnostic planar 131I-WBS after rh-TSH stimulation, in both anterior and posterior views (**a**, **b**), and planar neck/thorax anterior and posterior views (**c**, **d**), do not show any foci of pathologic radioiodine uptake. SPECT/CT in coronal (**e**, **f**), sagittal (**g**, **h**) and transverse (**i**, **l**) fusion images evidenced one radioiodine-avid focus (white arrows) in right laterocervical region adjacent to the sub-mandibular gland, in a posterior position, classifying it as lymph node metastasis also visible at CT images. Thyroglobulin levels were undetectable in basal conditions reaching values of 7.8 ng/ml after rh-TSH stimulation; anti-thyroglobulin antibodies were negative. Thus, the patient underwent a second therapeutic dose of radioiodine

Figure 3 shows the location of the 93 neck LN metastases ascertained at SPECT/CT and of the 34/93 nodes also evidenced at WBS, the latter without a clear characterization with the planar procedure. Moreover, all LN metastases adjacent to sub-mandibular glands were ascertained only by SPECT/CT (a case is illustrated in Fig. 4).

**Fig. 5:**
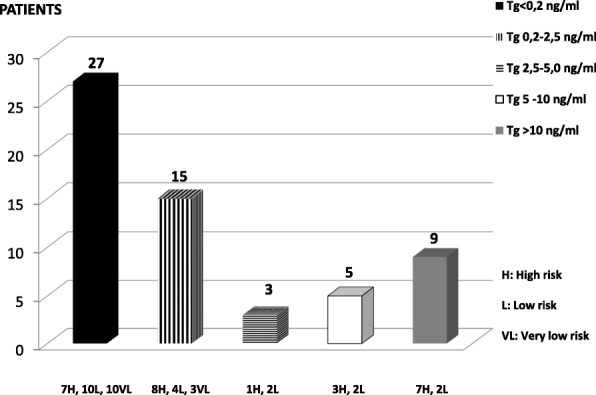
DTC patients with neck lymph node metastases (59 cases) subdivided into different groups according to thyroglobulin serum levels

According to thyroglobulin levels, the 59 patients with neck LN metastases were subdivided into different groups, as shown in Fig. 5.

Moreover, all the 13 VL patients (5 of whom with LN metastases adjacent to submandibular glands) with thyroglobulin levels < 0.2 ng/ml (10 cases) or < 2.5 ng/ml (3 cases) were T1aN0M0.

In total, with SPECT/CT, the LN status changed from N0 at the surgery to N1 during follow-up in 46 of the 59 cases. In 13/46 cases, SPECT/CT changed the risk classification from VL to L. The follow-up after surgery was at least 12 months but not more of 96 months, and the range time of LN metastasis appearance after the initial tumor diagnosis was 8–57 months.

Globally, SPECT/CT obtained an incremental value in respect of WBS in 45.5% of patients considering the identification of more thyroid tissue residues and neck metastatic lesions. A more correct patient classification changing therapeutic approach there was in 30.3% of cases; the possibility of avoiding unnecessary therapies in presence of single foci of physiologic iodine uptake not correctly classified at WBS was observed in 8% of patients.

## Discussion

Despite controversial significance, the presence of neck LN metastases in DTC seems more frequently to affect disease prognosis since the affected patients already at surgery may more likely undergo recurrences or metastases in the follow-up. Moreover, a high LN ratio, defined as the number of metastatic LN divided by the number of removed LN, and the presence of LN macro metastases seem to have a significant prognostic value [37]. Thus, it is crucial to correctly evaluate patient staging after thyroidectomy and radioiodine ablation therapy to permit an adequate monitor of patients in a long-term follow-up for re-staging the disease and appropriately changing the therapeutic strategy.

In the last years, ^131^I-SPECT/CT proved a reliable diagnostic tool to accurately identify and characterize neck LN metastases in DTC patients in both post-thyroidectomy pre-ablation phase [24, 26, 38], and in post-radioiodine therapy phase [21, 28–33]. The procedure substantially reduced the number of occult and unclear radioiodine avid foci at planar WBS; the latter has been considered for many years the reference imaging method in the diagnostic protocol of DTC after thyroidectomy. SPECT/CT can also determine LN status in the neck more accurately than planar WBS [16, 19, 26–30], also identifying rarer parapharyngeal metastases [39].

In the present study performed in DTC patients already submitted to thyroidectomy and radioiodine ablation and in long-term follow-up, SPECT/CT with diagnostic radioiodine dose proved a reliable tool to better identify, precisely localize and characterize neck ^131^I-avid foci compared to planar WBS. SPECT/CT also detected more iodine-avid foci than WBS allowing the identification of additional occult not-suspicious cervical LN metastases not evidenced by WBS and clarified unclear images or wrongly classified foci, as also reported by other authors [16, 40, 41]. Regarding this latter aspect, planar WBS localized foci in thyroid bed classifying these as remnant tissue in some cases, while the foci corresponded to cervical LN because of the poor anatomic information provided by planar procedure; however, image interpretation on SPECT/CT was more accurate, improving WBS diagnostic specificity. Among unclear foci, there were also areas of physiologic uptake, to the interpretation of which SPECT/CT contributed to clarifying false-positive results of WBS; this result was even more important when these foci were single, for avoiding unnecessary treatments.

Thus, the performance of WBS was improved for the diagnosis, staging, and follow-up in patients with neck LN metastases contributing to correctly change patient classification and management, including surgery or radioiodine therapies, and avoiding additional imaging procedures and guiding the decision on long-term follow-up strategy.

In particular, the data obtained in the present study demonstrated that SPECT/CT gave more information on LN staging in an elevated percentage of patients, thus resulting in a completion of TNM staging and risk new stratification. SPECT/CT superiority than planar WBS, that is in agreement with previous results obtained by some authors [16, 19, 27–30], is particularly significant when the latter is inconclusive and thyroglobulin levels are undetectable or very low in VL cases with T1aN0M0, and even more with single LN metastasis. Moreover, SPECT/CT performance was even higher than WBS when the nodes are sited in regions where these can be difficult to be discriminated from thyroid remnants or physiologic structures with elevated iodine uptake; among these, there are those adjacent to sub-mandibular glands that can affect the identification of neoplastic nodes. The latter were detected by SPECT/CT but not by WBS in our patients. However, as a rule, a microscopic disease in LN may escape detection by SPECT/CT like WBS.

The results of this study also confirmed that thyroglobulin levels could remain undetectable or very low in presence of neck LN metastases, particularly when these are single and the neck represents the only site of recurrence, thus suggesting that undetectable thyroglobulin levels cannot exclude a metastasis in an LN. Also in these cases, SPECT/CT proved useful to change risk stratification and treatment.

In the present study, the multifocal disease had been ascertained at the surgery in 40.7% of PC patients who developed metastases during long-term follow-up suggesting that these cancers have an increased metastatic potential, as reported by other authors [6], also including papillary microcarcinoma [19]. Moreover, 24.1% of PC patients also had lymph node metastases already at surgery as well as 14.8% of cases had extra-thyroid tumor extension, these disorders representing important risk factors for neck recurrences during follow-up, in particular when cervical nodes are numerous and even of large volume [37, 42–45]. Furthermore, the latter two factors were also associated with multifocal disease in some cases and, besides, in 13.5% of PC patients all three factors were present with a higher possibility of disease progression. Moreover, unlike the results reported by other authors [6], the size of the primary tumors in our cases seems to have a significant effect on neck LN metastasis appearance in the follow-up, 61% of patients having carcinomas > 10 mm.

Based on the present data on a long-term follow-up of a large series of DTC patients with radioiodine avid foci only in the neck,^131^I-SPECT/CT proved to add an important contribution to planar WBS interpretation. These favorable results agree with the data obtained in previous studies [16, 19], also in both pre-ablation and post-therapeutic phases [25–30, 46].

However, a relative limitation due to the retrospective nature of the present study should be considered.

The presence of very small size nodes, which can also be the site of microscopic metastases, could cause problems of identification associating the diagnostic dose of radioiodine with the low spatial resolution of the machine by partial volume effect. However, microscopic metastases do not seem to significantly get worse disease prognosis and only histology could give a correct definition since no imaging procedure can give certain information.

Furthermore, any misregistration defects have been limited by immobilizer systems utilized during exam acquisition, which, however, results in longer duration with possible patient discomfort.

Concerning radiation additional dose from CT to that of radioiodine, there is not an elevated exposure from low energy CT with effective doses for neck/chest of one mSv in average with the machine used in present study and in other studies [47]; such a slight exposure can be considered negligible considering the benefits of SPECT/CT technology.

The results of the present study cannot be generalizable being obtained in only one center, and the experienced nuclear medicine physicians who independently interpreted the SPECT/CT images belonged to the same Department.

The patients submitted to SPECT/CT and WBS have been enrolled in part in hypothyroidism after hormone therapy interruption and in part after rhTSH stimulation. Moreover, some LN metastases detected by SPECT/CT have not confirmed by histopathologic examinations for practical and ethical reasons, but only validated by follow-up with clinical data, thyroglobulin variations and the results of radiologic and nuclear medicine procedures.

Finally, being DTC carcinomas slowly growing malignancies, these can require a more prolonged follow up since some foci classified as benign might prove metastases late as well as eventual micrometastasis in a lymph node, that may be occult at the first examination of SPECT/CT like WBS, might be revealed macrometastasis afterward. A more prolonged follow up might also permit a better evaluation of the outcome of therapeutic strategies.

Despite its limitations, SPECT/CT in this study proved to be a useful diagnostic imaging procedure giving a better characterization of focal areas of uptake excluding malignancy in physiologic sites of tracer uptake. The procedure obtained a more correct anatomic localization of the lesions, thus helping planar WBS interpretation and demonstrated an elevated impact on patient management and therapy planning. SPECT/CT is a simple and non-invasive method that gave in all cases high-quality images that were easy to interpret.

## Conclusions

Based on the results of the present study, we can suggest that ^131^I- SPECT/CT with diagnostic dose should be used routinely, as a complementary tool to planar WBS, in the evaluation of patients with differentiated thyroid carcinoma in long term follow-up to early identify neck lymph node metastases. Post-surgical very low-risk patients and T1a N0M0 cases should also be included as well as those with unclear findings at WBS, and even when the latter is negative and thyroglobulin is undetectable or very low. SPECT/CT can be able to detect more iodine avid neck foci than WBS and to differentiate LN metastasis from remnant thyroid tissue or areas of benign or physiologic uptake, thus reducing unclear and false-positive results. The procedure contributed to guide the most appropriate treatment of affected patients and proved to be more helpful for calculating the therapeutic dose of radioiodine.

Moreover, a long-term follow-up is necessary to better guarantee careful surveillance of affected patients considering that differentiated thyroid carcinoma has slow growth and it is not possible to predict when an occult metastasis will become clinically evident. In the next future, ^131^I-SPECT/CT will have ever-growing importance and will become progressively more indispensable in the management of patients with DTC to early ascertain recurrences and in particular neck LN metastases in the course of long-term follow-up together with neck ultrasound and serum thyroglobulin assay that are standard procedures included in the diagnostic protocol of DTC patients.

## Data Availability

The dataset used and/or analyzed during the current study are available from the corresponding author on reasonable request.

## Abbreviations

DTC: Differentiated thyroid carcinoma
LN: Lymph node
WBS: Whole body scan
H: High risk
L: Low risk
VL: Very low risk
PC: Papillary carcinoma
FC: Follicular carcinoma
HCC: Hürthle cell carcinoma
TSH: Thyroid-stimulating hormone
rhTSH: Recombinant human TSH
Ab-Tg: Anti-thyroglobulin antibodies
